# Sleep quality assessment in hospitalized postoperative surgical patients: a COSMIN-based systematic review

**DOI:** 10.3389/frsle.2025.1714777

**Published:** 2026-04-13

**Authors:** Nunung Nurhayati, Agung Waluyo, I. Made Kariasa, Sali Rahadi Asih, Hening Pujasari, Bahrul Hayat

**Affiliations:** 1 Doctoral Study Program in Nursing, Faculty of Nursing, University of Indonesia, Depok, Indonesia; 2 Department of Critical and Emergency Nursing, Sekolah Tinggi Ilmu Keperawatan Persatuan Perawat Nasional Indonesia (PPNI) Jawa Barat, Bandung, Indonesia; 3 Department of Medical Surgical Nursing, Faculty of Nursing, Universitas Indonesia, Depok, Indonesia; 4 Faculty of Psychology, University of Indonesia, Depok, Indonesia; 5 Department of Basic Science and Fundamental of Nursing, Faculty of Nursing, University of Indonesia, Depok, Indonesia; 6 Department of Psychology, Faculty of Psychology, Universitas Islam Negeri Syarif Hidayatullah Jakarta, East Jakarta, Indonesia

**Keywords:** COSMIN, postoperative surgical patients, psychometric properties, sleep quality, sleep assessment tools, systematic review

## Abstract

**Background:**

Sleep is a crucial physiological process that significantly influences recovery among hospitalized postoperative surgical patients, including those treated in intensive care units and general surgical wards. Reliable and valid instruments for assessing sleep quality are essential for guiding clinical decision-making and improving patient outcomes. However, the psychometric properties of commonly used sleep assessment tools remain inconsistent, highlighting the need for systematic evaluation.

**Objective:**

This study aimed to conduct a COSMIN-based systematic review to examine the measurement properties of sleep quality assessment instruments used in hospitalized postoperative surgical patients.

**Methods:**

A systematic literature search was conducted across PubMed, Scopus, Web of Science, CINAHL, and PsycINFO for studies published between 2010 and 2024. The methodological quality of each study was evaluated using the COSMIN Risk of Bias checklist, and a narrative synthesis was performed to summarize the psychometric evidence for each instrument.

**Results:**

Of the 210 studies initially identified, 37 met the eligibility criteria. The Pittsburgh Sleep Quality Index (PSQI) demonstrated adequate reliability and construct validity but showed limitations related to measurement error and responsiveness. The Richards–Campbell Sleep Questionnaire (RCSQ) exhibited strong reliability and construct validity, though variability was observed in interrater agreement between nurses and patients. The Epworth Sleepiness Scale (ESS) was reliable for assessing daytime sleepiness but provided limited evidence for structural validity in postoperative contexts. The Sleep Quality Questionnaire (SQQ), Verran and Snyder-Halpern Sleep Scale (VSH), and Insomnia Clinical Evaluation (ICE) showed mixed psychometric properties, indicating the need for further validation in hospitalized postoperative populations.

**Conclusion:**

The PSQI and RCSQ remain the most frequently utilized sleep assessment instruments; however, their psychometric limitations warrant cautious interpretation. This review underscores the need for further research to refine, validate, and potentially develop more robust sleep assessment tools tailored to hospitalized postoperative surgical patients.

## 1 Introduction

Sleep is a fundamental physiological process that plays a crucial role in postoperative recovery and overall patient wellbeing. Among hospitalized postoperative surgical patients, including those admitted to intensive care units (ICUs) and general surgical wards, sleep disturbance is highly prevalent due to factors such as pain, environmental noise, anxiety, pharmacologic effects, and underlying medical conditions (Yilmaz et al., 2012). In addition to environmental and clinical stressors, patient-related metabolic and endocrine characteristics, such as prediabetes/diabetes, obesity, and insulin resistance are increasingly recognized as contributors to altered sleep architecture and sleep fragmentation in postoperative or critically ill patients (Dutil et al., 2024). For instance, people with diabetes or prediabetes show reduced REM sleep and shorter total sleep time even in the absence of severe sleep apnea (Chen et al., 2024), and in obese adolescents at risk of type 2 diabetes, increasing sleep duration improved insulin sensitivity (Yi et al., 2009).

Sleep disturbance has wide-ranging physiological, cognitive, and psychosocial consequences. Clinically, inadequate postoperative sleep is linked to delayed wound healing, impaired immune function, higher rates of complications, prolonged hospitalization, and poorer physical and cognitive recovery trajectories (Boyko et al., 2017). Neurocognitively, disrupted sleep impairs the processes of memory retention and consolidation, which depend on the reactivation of neural traces during slow-wave sleep and their integration into long-term storage networks (de Blacam et al., 2024; Nema et al., 2024; Prakrithi et al., 2019). Patients with disrupted sleep also report increased headaches, lower satisfaction with life, diminished emotional regulation, and poorer general health perception, further compounding recovery challenges (Meira E Cruz and Andersen, 2025; Orzeszek et al., 2025; Seid Tegegne and Fenta Alemnew, 2022; Selçuk Sert et al., 2024). Sleep disruption is also linked to broader metabolic dysregulation, including impaired glucose homeostasis, inflammation, and cardiometabolic risk (St-Onge et al., 2023).

Sleep difficulties are highly prevalent among hospitalized postoperative surgical patients, including those receiving care in intensive care units and general surgical wards. Multiple factors contribute to sleep disturbance in this population, such as ambient noise, postoperative pain, medication effects, and underlying medical conditions (Kamdar et al., 2012). Poor sleep quality in hospitalized postoperative patients has been linked to delayed recovery, increased morbidity, and extended hospital stays (Boyko et al., 2017). Despite its recognized importance, sleep quality in postoperative surgical settings remains insufficiently examined, and the instruments used to assess sleep often demonstrate inconsistent performance or lack adequate validation for this patient group. Understanding sleep quality in hospitalized postoperative patients is essential, as the interaction between the hippocampus and retrosplenial cortex during rapid eye movement (REM) sleep enhances memory recall through theta-driven connectivity (Dos Santos Bento et al., 2023). In addition, slow-wave sleep plays a crucial role in memory consolidation, with slow oscillations and sleep spindles supporting the retention of newly acquired information (Scott et al., 2021).

A review of the literature shows that sleep quality among postoperative surgical patients is frequently assessed using subjective tools such as the Richards–Campbell Sleep Questionnaire (RCSQ) and the Pittsburgh Sleep Quality Index (PSQI), alongside objective measures like polysomnography (PSG) and actigraphy (Altman et al., 2018). Although PSG remains the gold standard for sleep measurement, its use in hospitalized postoperative environments is limited due to financial cost, operational complexity, and patient discomfort (Delaney et al., 2021). While subjective instruments offer a more feasible alternative, many lack rigorous validation in postoperative or critical-care contexts, raising questions about their reliability and validity (Jeffs and Darbyshire, 2019). The diversity of available tools and the variability in their psychometric quality further complicate cross-study comparisons (Darbyshire et al., 2020). Earlier evaluations, such as that by Beecroft et al. (2008), identified the RCSQ as a promising subjective instrument, yet emphasized the need for stronger psychometric evidence. Selecting appropriate patient-reported outcome measures (PROMs) therefore requires comprehensive assessment of their measurement properties.

A thorough analysis of current sleep assessment instruments reveals substantial A deeper analysis of current sleep assessment tools reveals substantial gaps in their development and validation. Many instruments were not originally designed for postoperative surgical populations, and their psychometric properties, specifically reliability, validity, and responsiveness remain insufficiently examined in both ICU and non-ICU postoperative settings (Friese, 2008). This lack of standardized, robustly validated instruments highlights the need for a systematic review that evaluates their suitability for hospitalized postoperative patients. The Consensus-based Standards for the Selection of Health Measurement Instruments (COSMIN) framework provides a rigorous, structured approach for evaluating key psychometric attributes such as validity and reliability (Mokkink et al., 2024). A COSMIN-based systematic review is therefore appropriate for this topic, as it enables a comprehensive and critical appraisal of existing tools, ensuring that only instruments with strong psychometric foundations are recommended for clinical and research use.

Although numerous tools are available to assess sleep quality in postoperative surgical settings, systematic evaluations of their measurement properties using COSMIN criteria are scarce. This gap underscores the need for a thorough and standardized examination of available instruments to inform evidence-based assessment and clinical decision-making. Accordingly, the present study aims to conduct a COSMIN-based systematic review to investigate the psychometric properties of tools used to evaluate sleep quality in hospitalized postoperative surgical patients. The objective is to identify and critically evaluate these instruments to determine which are most reliable and valid for use in clinical practice and future research.

## 2 Materials and methods

### 2.1 Study design

This study employed a systematic review approach to assess the quality of sleep assessment tools used among hospitalized postoperative surgical patients, including those in intensive care units and general surgical wards. The review followed the Preferred Reporting Items for Systematic Reviews and Meta-Analyses (PRISMA) guidelines (Page et al., 2021) and adhered to the COSMIN (Consensus-based Standards for the Selection of Health Measurement Instruments) methodology to ensure a rigorous and standardized evaluation of the measurement properties of the identified instruments (Terwee et al., 2015).

### 2.2 Search strategy

A comprehensive literature search was conducted across five electronic databases: PubMed, Scopus, Web of Science, CINAHL, and PsycINFO. The search covered studies published in English between January 2010 and December 2024. To enhance reproducibility and transparency, the search strategy combined Medical Subject Headings (MeSH) and free-text keywords related to *sleep quality, postoperative or surgical patients, intensive care units (ICU), sleep assessment tools*, and *measurement properties*. Boolean operators (AND/OR) and truncation symbols (e.g., ^*^) were used to refine and broaden the search. The search string was used in PubMed and can be replicated exactly (Box 1).

Box 1 **Example of a full search string (PubMed).**

(“Sleep”[Mesh] OR “Sleep Wake Disorders”[Mesh] OR “Sleep Quality”[tiab] OR “sleep disturbance”[tiab]

OR “sleep disorders”[tiab] OR “sleep pattern”[tiab])

AND

(“Intensive Care Units”[Mesh] OR “Postoperative Care”[Mesh]

OR “postoperative”[tiab] OR “post-operative”[tiab] OR “surgical patients”[tiab]

OR “post-surgery”[tiab] OR “after surgery”[tiab])

AND

(“Psychometrics”[Mesh] OR “Validation Studies as Topic”[Mesh]

OR “measurement properties”[tiab] OR “psychometric”[tiab]

OR “reliability”[tiab] OR “validity”[tiab] OR “instrument”[tiab]

OR “assessment tool”[tiab] OR “questionnaire”[tiab])

AND

(“2010/01/01”[Date - Publication] : “2024/12/31”[Date - Publication])

This search string was adapted for other databases by replacing MeSH terms with the respective subject headings in CINAHL and PsycINFO while retaining the same free-text terms and Boolean structure. In Scopus and Web of Science, which do not use controlled vocabulary, the strategy relied entirely on free-text keywords with the same logical arrangement. To enhance completeness, the reference lists of all included articles and relevant reviews were also screened to identify additional eligible studies. This approach ensures that the overall search process is transparent, systematic, and fully reproducible.

### 2.3 Inclusion and exclusion criteria

The inclusion criteria were clarified to ensure that studies evaluating the measurement properties of sleep assessment tools in hospitalized postoperative surgical patients—including those admitted to intensive care units, high-dependency units, and general surgical wards—were eligible regardless of whether they explicitly applied COSMIN methodology. The intention was not to restrict inclusion only to studies that mentioned COSMIN, but to include any research that reported psychometric data such as validity, reliability, or responsiveness. Studies that lacked such measurement information were excluded. To improve search completeness and avoid missing relevant studies, the search strategy was expanded to include terms related to measurement evaluation, such as “psychometric,” “measurement properties,” “instrument validation,” and “reliability,” rather than relying solely on the term “COSMIN,” which is inconsistently used in the literature. This approach ensured that studies aligned with COSMIN principles, even if not explicitly labeled as such, were captured.

Studies were excluded if they did not focus on postoperative surgical populations, lacked psychometric data, were not published in English, were not original research (such as reviews, commentaries, conference abstracts), or lacked full-text availability or sufficient methodological quality. These revisions ensure a clear and transparent linkage between the search strategy, screening decisions, and the final set of included studies.

### 2.4 Data extraction

Two independent reviewers extracted data using a standardized data extraction form. Information collected included study characteristics (authors, year, country, sample size, and study design), details of sleep assessment instruments, measurement properties, and key findings. Discrepancies were resolved through discussion or consultation with a third reviewer.

### 2.5 Quality assessment

The methodological quality of included studies was assessed using the COSMIN Risk of Bias checklist (Terwee et al., 2015). The methodological quality of each included study was assessed using the COSMIN Risk of Bias checklist, which evaluates domains such as content validity, construct validity, reliability, responsiveness, and measurement error. COSMIN does not use numerical scores or quantitative cut-off ranges to determine whether a study is “very good,” “adequate,” “doubtful,” or “inadequate.” Instead, each rating is assigned based on whether the study meets the methodological standards specified for each item within a measurement property. An item is rated “very good” when it fully satisfies the COSMIN criteria, “adequate” when the methods are acceptable but not optimal, “doubtful” when important methodological aspects are unclear or insufficiently reported, and “inadequate” when the study clearly fails to meet established standards. Consistent with COSMIN rules, the overall rating for each measurement property reflects the lowest rating among its items, following the “worst-score counts” principle. The certainty of the body of evidence was subsequently evaluated using the GRADE approach, which assesses four key factors: risk of bias, inconsistency, imprecision, and indirectness. Risk of bias reflects methodological limitations identified through the COSMIN appraisal. Inconsistency refers to variability in findings across studies evaluating the same measurement property. Imprecision considers the width of confidence intervals and sample sizes affecting the reliability of the estimates. Indirectness evaluates the extent to which the study populations, settings, or outcomes differ from the review question. Based on these domains, the overall strength of evidence for each measurement property was rated as high, moderate, low, or very low (Schünemann et al., 2019).

### 2.6 Data analysis

A narrative synthesis was performed to summarize the measurement properties of identified sleep assessment tools. The COSMIN framework was used to classify and interpret findings based on predefined criteria.

## 3 Results

### 3.1 Searching results

**Figure 1:**
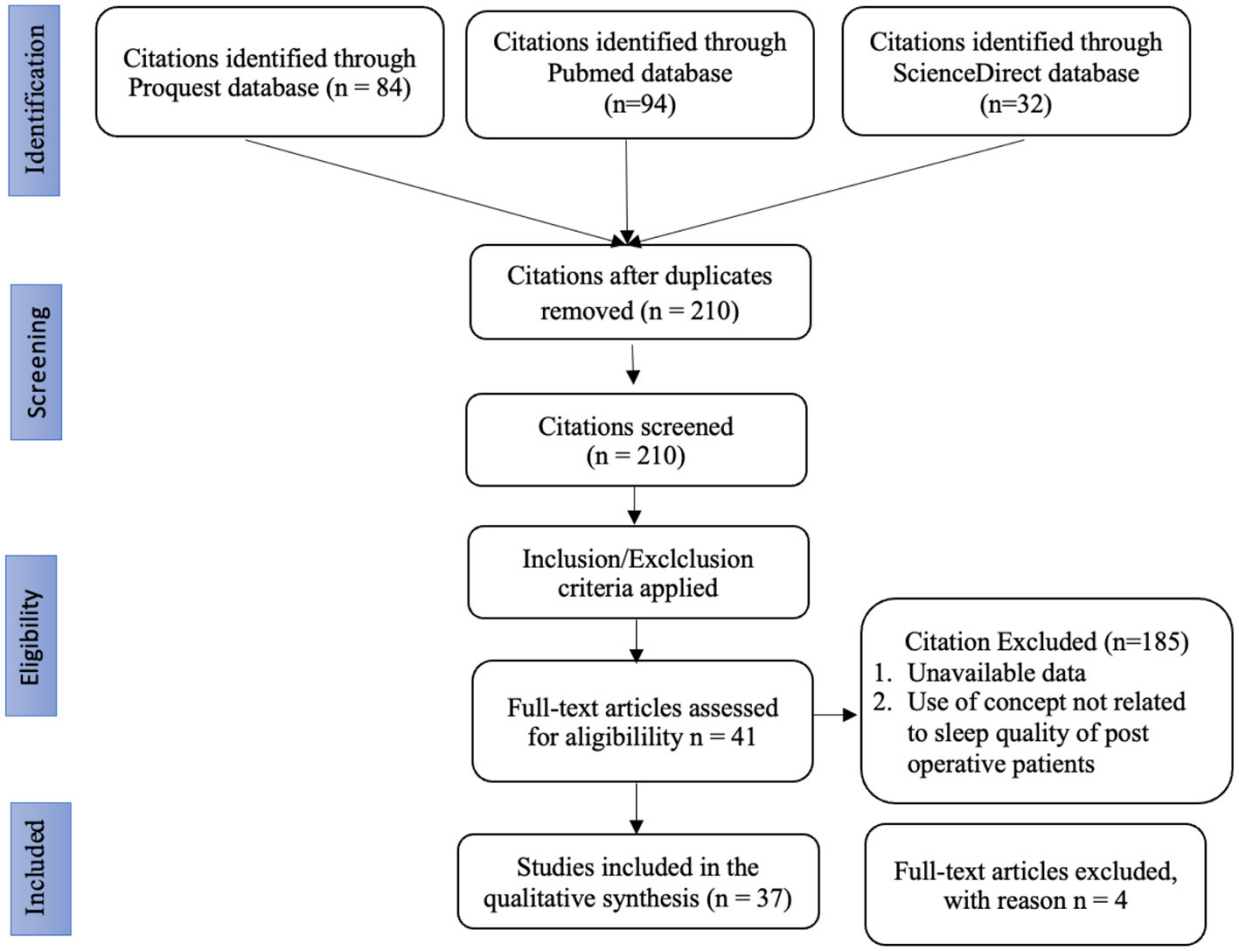
PRISMA flow diagram.

A total of 210 records were screened after duplicates were removed. Following title and abstract screening, 169 studies were excluded for not meeting the eligibility criteria, leaving 41 articles for full-text review. Of these, four articles were excluded due to unavailable full text or lack of relevance to postoperative sleep assessment. Ultimately, 37 studies met the inclusion criteria and were incorporated into the COSMIN-based evaluation. The full selection process is illustrated in the PRISMA flow diagram (Figure 1). A detailed list of all 37 included studies, including clinical setting (ICU, step-down, or general surgical ward) and notes on postoperative relevance, is provided in Supplementary Table S1.

### 3.2 Patient-reported sleep measures in postoperative and ICU populations

Table 1 provides a summary of the instruments utilized across the included studies, along with their respective authors and the number of studies in which each instrument was applied. The Pittsburgh Sleep Quality Index (PSQI) emerged as the most frequently used instrument, appearing in 19 studies, followed by the Richard–Campbell Sleep Questionnaire (RCSQ), which was employed in 10 studies. The Epworth Sleepiness Scale (ESS) was used in five studies, whereas the Sleep Quality Questionnaire (SQQ), Verran and Snyder-Halpern Sleep Scale (VSH), and Insomnia Clinical Evaluation (ICE) were each used in only one study.

**Table 1:** The summary of the included studies.

Name of instruments	Authors/year	No. of studies
PSQI	Seid Tegegne and Fenta Alemnew, 2022; Selçuk Sert et al., 2024; Prakrithi et al., 2019; Yilmaz et al., 2012; Büyükyilmaz et al., 2011; Barichello et al., 2009; Telias and Wilcox, 2019; Solverson et al., 2016; Oren et al., 2020; Leong et al., 2021; Okkesim et al., 2019; Yildiz et al., 2021; Chen et al., 2016; Luo et al., 2019; Bang and Park, 2020; Grubbs et al., 2023; Díaz-Alonso et al., 2018; Wang et al., 2020; Hu et al., 2022	19
RCSQ	Navarro-García et al., 2017; Allen et al., 2022; Rood et al., 2019; Nagatomo et al., 2020; Tonna et al., 2021; Alsulami et al., 2019; Fazlollah et al., 2021; Grubbs et al., 2023; Díaz-Alonso et al., 2018; Wang et al., 2020	10
SQQ	Myoji et al., 2015	1
ESS	Chen et al., 2016; Luo et al., 2019; Bang and Park, 2020; Bakry et al., 2022; Hu et al., 2022	5
VSH	Khoddam et al., 2022	1
ICE	Li J. et al., 2022	1

PSQI, The Pittsburgh Sleep Quality Index; RCSQ, the Richard-Campbell Sleep Questionnaire; ESS, the Epworth Sleepiness Scale; SQQ, the Sleep Quality Questionnaire; ICE, insomnia Clinical Evaluation; VSH, Verran and Snyder-Halpern.

#### 3.2.1 The Pittsburgh Sleep Quality Index

Across the included studies, the PSQI was used to assess global sleep quality in postoperative patients after major oncologic, orthopedic, abdominal and cardiothoracic procedures, most often in surgical wards or mixed ICUs rather than in exclusively medical populations (Barichello et al., 2009; Büyükyilmaz et al., 2011; Pröpper et al., 2015; Seid Tegegne and Fenta Alemnew, 2022; Selçuk Sert et al., 2024).

The PSQI consists of a 4-point Likert scale, where each sleep component is scored from 0 to 3, with 3 indicating the highest level of dysfunction. Individual component scores are summed to generate a global score ranging from 0 to 21, with higher scores indicating poorer overall sleep quality (Barichello et al., 2009). The key components assessed by the PSQI include sleep duration, sleep disturbances, sleep latency, daytime dysfunction due to sleepiness, sleep efficiency, overall sleep quality, and sleep medication use (Barichello et al., 2009). Despite its widespread use and validation, the PSQI does not comprehensively cover all dimensions of sleep health (Tonna et al., 2021).

The PSQI is widely used to differentiate between individuals with high and low sleep quality, with a commonly applied cut-off score of 5 (Tsai et al., 2022; Qin et al., 2020). The PSQI's diagnostic accuracy for sleep disorders varies; it has shown high sensitivity and specificity for detecting insomnia in certain populations (Büyükyilmaz et al., 2011) but has demonstrated limited accuracy in identifying other sleep disorders (Barichello et al., 2009). Some studies have reported a weak correlation between PSQI scores and objective sleep measures, such as polysomnography (Ghani et al., 2020), while others have found significant correlations with sleep diary variables (Okkesim et al., 2019).

The PSQI is an effective tool for distinguishing between individuals with good and poor sleep quality (Busija et al., 2020) and has demonstrated good internal consistency in elderly male patients (Pratiwi et al., 2021). Notably, gender differences in PSQI scores have been observed, with women reporting poorer sleep quality than men (Leong et al., 2021). However, caution is advised when interpreting PSQI scores due to potential overlap with measures of depression, anxiety, and stress (Nagatomo et al., 2020). Some studies have reported significant correlations between PSQI scores and these psychological factors (Sheng et al., 2021; Qin et al., 2020), suggesting that the PSQI may reflect symptoms of depression or negative cognition rather than objectively measuring sleep quality (Ghani et al., 2020). In particular, PSQI scores often correlate strongly with measures of depression and anxiety (Ghani et al., 2020), indicating that the scale may capture general dissatisfaction rather than specific sleep disorders.

The PSQI has been translated and validated in multiple languages, including Urdu and Indonesian, demonstrating strong linguistic adaptability (Al-Habsi et al., 2022). It has also been validated in Arabic and Moroccan dialects (Guerra et al., 2023). Additionally, versions of the PSQI have been successfully validated in Spain, Korea, Serbia, and Thailand, confirming its psychometric robustness across different cultural contexts (Kwak et al., 2020). The PSQI has also shown acceptable internal consistency and reliability across various cultural contexts, including populations in India (Al-Mughales et al., 2022) and the Netherlands (Becking-Verhaar et al., 2023), as well as among adolescents (Rood et al., 2019). The PSQI has also been validated in specific populations, including cancer patients, elderly men, pregnant women, and individuals living with HIV (Qiu et al., 2022).

From a COSMIN perspective, internal consistency was acceptable to good in most studies, with Cronbach's α ranging from 0.61 to 0.89 across diverse samples, including older adults and patients with chronic conditions (Gomaa et al., 2022; Buysse et al., 1989; Hsu et al., 2021). Test–retest reliability was adequate, with intraclass correlation coefficients (ICC) between 0.76 and 0.997 in repeated assessments (Molaee et al., 2023). Convergent validity was supported by moderate to strong correlations with other sleep measures and related constructs such as depression, anxiety and fatigue, although these high correlations also indicate construct overlap with psychological distress rather than pure sleep parameters (Ghani et al., 2020; Sheng et al., 2021; Qin et al., 2020). Evidence on structural validity and responsiveness in postoperative ICU cohorts remains sparse; most studies reported pre–post changes in mean PSQI scores without formal responsiveness indices. Overall, PSQI shows at least moderate evidence of sufficient internal consistency and reliability, but limited, indirect evidence for validity and responsiveness specifically in postoperative ICU patients.

#### 3.2.2 The Richard-Campbell Sleep Questionnaire

The RCSQ was the only instrument specifically developed and most consistently validated for ICU use. It was applied in surgical and mixed ICUs after major abdominal, cardiothoracic, and orthopedic procedures, as well as in general critical care cohorts (Myoji et al., 2015; Delaney et al., 2022; Tonna et al., 2021; Varella et al., 2021; Locihová et al., 2020). In these settings, the RCSQ captured depth of sleep, sleep latency, awakenings and overall sleep quality using 0–100 mm visual analog scales (Masa et al., 2020).

The questionnaire has shown moderate to strong correlations with other sleep measures and effectively distinguishes between individuals with good and poor sleep quality (Myoji et al., 2015). The RCSQ is also associated with sleep continuity, a factor linked to positive health outcomes in patients (Delaney et al., 2022). Studies indicate that sleep quality and responsiveness to external stimuli vary across sleep stages, with deeper sleep stages associated with reduced responsiveness (Luo et al., 2019). Additionally, age-related changes in responsiveness during sleep have been observed, particularly in infants (Bang and Park, 2020).

In contrast to the Pittsburgh Sleep Quality Index (PSQI), the Richards-Campbell Sleep Questionnaire (RCSQ) is a simple and validated tool for assessing sleep quality in intensive care unit (ICU) patients, with Cronbach's alpha values ranging from 0.88 to 0.96. The RCSQ demonstrates strong construct and concurrent validity, making it a reliable tool for assessing sleep quality in both ICU patients and individuals sleeping at home (Ritmala-Castrén et al., 2022). It effectively differentiates between sleep quality in ICU settings and home environments (Ritmala-Castrén et al., 2022). However, the interrater reliability between patients and nurses using the RCSQ has been found to be “slight” to “moderate,” with nurses tending to overestimate the quality of sleep perceived by patients (Tonna et al., 2021). Other studies have demonstrated its reliability and validity in Brazilian Portuguese (Varella et al., 2021; Jolley et al., 2018), as well as in China and the Netherlands (Becking-Verhaar et al., 2023).

According to COSMIN criteria, the RCSQ demonstrated high internal consistency, with Cronbach's α typically between 0.88 and 0.96 in ICU samples (Ritmala-Castrén et al., 2022; Varella et al., 2021). Construct and concurrent validity were supported by moderate to strong correlations with other subjective sleep ratings and by its ability to distinguish between ICU and home sleepers (Ritmala-Castrén et al., 2022; Delaney et al., 2022). Cross-cultural adaptations (e.g., Brazilian Portuguese, European cohorts) retained the original factor structure and showed comparable reliability (Varella et al., 2021; Fazlollah et al., 2021). However, agreement with objective measures (actigraphy or polysomnography) was only fair, with some studies reporting low concordance between RCSQ scores and activity-based indices (Locihová et al., 2018). Inter-rater reliability between nurses and patients was “slight” to “moderate,” with nurses tending to overestimate patient sleep quality (Tonna et al., 2021). Responsiveness data are limited but suggest that the RCSQ can detect changes after non-pharmacological sleep interventions in ICU (Karimi et al., 2021; Shih et al., 2022). Overall, the RCSQ provides strong evidence for internal consistency and construct validity and moderate evidence for responsiveness in postoperative and ICU contexts, but nurse-proxy ratings should be interpreted with caution.

#### 3.2.3 The Sleep Quality Questionnaire

Evidence for the SQQ in postoperative or ICU populations is more indirect. Most psychometric evaluations were conducted in community, student or chronic disease cohorts, with only a minority of studies including postoperative or hospitalized patients (Sainsbury et al., 2021; Costa et al., 2022; Solverson et al., 2016). Across these settings, total scores range from 0 to 100, with higher scores indicating better sleep. The instrument assesses multiple dimensions of sleep, including: sleepiness upon waking, sleep initiation and maintenance, frequent dreaming, feeling refreshed upon waking, and sleep duration.

The SQQ has been validated across diverse populations, demonstrating strong psychometric properties. The research has consistently found the SQQ to be a valid and reliable tool for assessing sleep quality. However, studies indicate that self-reported sleep duration often overestimates actual sleep time, as measured by polysomnography or actigraphy (Costa et al., 2022). This discrepancy underscores the importance of considering measurement errors when analyzing sleep data (Lee et al., 2017). Studies conducted in China, Spain, Iran, Nigeria, and Turkey have reported that the SQQ exhibits strong internal consistency, with Cronbach's alpha ranging from 0.70 to 0.93, and high test-retest reliability (Zhang et al., 2024). Additionally, the SQQ demonstrates good construct validity, with factor analyses supporting a multi-dimensional structure (Zhang et al., 2024). The questionnaire also exhibits strong convergent validity, correlating well with other widely used sleep measures, such as the Pittsburgh Sleep Quality Index (PSQI) (Sainsbury et al., 2021). Furthermore, the SQQ effectively differentiates between clinical and non-clinical populations (Pröpper et al., 2015) and is sensitive to changes in sleep qualityover time (Hojjati et al., 2022). Similar psychometric properties have been reported for related sleep measures, such as the Sleep Quality Scale, particularly in patients with obstructive sleep apnea (Solverson et al., 2016).

In addition, the SQQ analyzer has been validated in diverse populations, demonstrating strong psychometric properties (Phillips and Hornacky, 2020). Studies have confirmed its structural validity, with a two-factor model providing the best fit (Sainsbury et al., 2021). Research involving medical students, Nigerian teenagers, and Chinese health students (Chen et al., 2022) has further validated the SQQ, confirming its reliability and validity. Moreover, the SQQ exhibits a strong correlation with other validated sleep measures, such as the PSQI (Dimick et al., 2023).

The SQQ demonstrates strong internal consistency, test-retest reliability, and convergent validity with other sleep measures (Chu et al., 2021). It also exhibits high specificity and sensitivity in identifying sleep problems (Akins et al., 2022). Other sleep questionnaires, such as the Pittsburgh Sleep Quality Index (PSQI) and the Quebec Sleep Questionnaire (QSQ), have also been validated across multiple languages and populations, demonstrating strong structural validity and internal consistency (Wang et al., 2021). Sleep questionnaires have been widely used to assess sleep quality across different groups, including individuals with obstructive sleep apnea and intellectual disabilities (Lee et al., 2017).

Interestingly, self-reported sleep quality has shown an inconsistent relationship with cognitive function, including reading comprehension (Ikeda et al., 2022). Sleep-dependent memory consolidation may influence language learning and sentence comprehension (Ikeda et al., 2022). Individuals with insomnia and normal sleep tend to define sleep quality similarly, emphasizing aspects such as daytime fatigue, feelings of rest, and nighttime awakenings (Bomfim, 2021). Moreover, sleep-disordered breathing (SDB) is associated with reduced vitality, while difficulty initiating and maintaining sleep (DIMS) and excessive daytime sleepiness (EDS) are strongly linked to poorer quality of life across multiple domains (Balk et al., 2023). Thus, while the SQQ appears psychometrically robust overall, there is insufficient direct evidence to recommend it as a primary outcome measure in postoperative ICU cohorts.

#### 3.2.4 The Epworth Sleepiness Scale

The ESS was used primarily to assess daytime sleepiness and risk of sleep-disordered breathing rather than nocturnal sleep quality in postoperative settings. It has been applied in perioperative cohorts with obstructive sleep apnea and bariatric surgery, as well as in broader medical populations (Suen et al., 2019; Bakry et al., 2022; Ortiz et al., 2024).

The ESS has been validated across various cultures and languages, demonstrating its effectiveness in measuring daytime sleepiness. Research has shown good internal consistency and reliability for translated versions in German (Merz et al., 2019), Arabic (Bakry et al., 2022), and Spanish (Sandoval, 2017). The ESS is widely regarded as a reliable and internally consistent self-report questionnaire for assessing daytime sleepiness (Ribbons et al., 2023). However, its clinical relevance and correlation with objective sleep measures vary across different populations. Studies have reported good internal consistency for the ESS, with Cronbach's alpha values ranging from 0.69 to 0.88 (Ortiz et al., 2024). Additionally, the ESS has demonstrated adequate internal consistency reliability (Cronbach's α = 0.70–0.81) and construct validity in diverse populations, including older men, pregnant women, and rural adolescents (Bauraite, 2024). However, concerns have been raised regarding the reliability of ESS retest scores, with some studies reporting low reliability over short time intervals (Heavner et al., 2023). Furthermore, in the Sleep Heart Health Study, inter- and intra-scorer reliability for sleep stage classification and respiratory distress index measurements were excellent, with kappa statistics exceeding 0.80 and intraclass correlation coefficients surpassing 0.90 for most measures (Oren et al., 2020). In postoperative ICU patients, the ESS has rarely been used and is not designed to capture environmental sleep disruption or nocturnal fragmentation. Consequently, its measurement properties for postoperative ICU sleep outcomes are largely unknown, and its role is better conceptualized as a complementary measure of residual daytime sleepiness rather than a core ICU sleep PROM.

#### 3.2.5 Verran and Snyder-Halpern

The VSH has been used in coronary care and postoperative cardiac surgery settings, where night-time environmental disruption is prominent (Khoddam et al., 2022; Jayanthi and Hudiyawati, 2019; Salamati et al., 2017). It captures sleep disturbance, effectiveness and supplementation, providing a more detailed profile of in-hospital sleep than single global scores.

The VSH is a validated instrument for measuring subjective sleep characteristics, demonstrating strong reliability and construct validity (Khoddam et al., 2022). Its validity has also been reinforced through cross-cultural adaptations, such as the Korean A Sleep Scale and the Persian version (Salamati et al., 2017). Similarly, the Sleep Quality Scale has shown high validity and reliability, particularly in individuals with obstructive sleep apnea syndrome (Voiriot et al., 2022). In sleep research, steady-state evoked potentials have been reliably detected in awake subjects but exhibit reduced amplitude and higher thresholds during sleep. However, self-reported sleep duration often overestimates actual sleep time when compared to objective measurements. Additionally, measurement errors in sleep studies may result from visual assessments of electrophysiological data and skewed distributions in sleep-disturbed respiratory measurements. Overall, the VSH has promising reliability and construct validity in cardiac and high-dependency settings, but direct evidence in heterogeneous postoperative ICU populations is still limited.

#### 3.2.6 Insomnia Clinical Evaluation

The ICE (with AIS thresholds often used for severity categorization) has been evaluated mainly in chronic insomnia, chronic pain, cancer, liver disease and other long-term conditions (Sun et al., 2021; Kawasaki et al., 2021; de la Varga-Martínez et al., 2023; Jaiswal et al., 2023). Postoperative and ICU applications are rare; most perioperative studies focus on insomnia prevalence rather than detailed validation of ICE in this context.

The ICE is a reliable and valid tool for assessing insomnia across diverse populations, including individuals with chronic pain (de la Varga-Martínez et al., 2023), cancer (Sun et al., 2021), and chronic liver disease (Kawasaki et al., 2021). The scale has been successfully adapted and validated in multiple languages, such as Taiwanese, Spanish, French, and Chinese (Wondmieneh, 2020). Studies consistently report strong psychometric properties, including high internal consistency, test-retest reliability, and concurrent validity. Severity criteria for the Athens Insomnia Scale (AIS) categorize insomnia into four levels: absent (0–5), mild (6–9), moderate (10–15), and severe (16–24) (Oka et al., 2010). The ICE has been linked to various health-related factors, including quality of life, depression, and work productivity (Jaiswal et al., 2023). Poor sleep quality, as assessed by the ICE, has been associated with negative health outcomes such as impaired weight loss maintenance, increased rheumatoid arthritis disease activity, and reduced overall quality of life and productivity. Nevertheless, given the scarcity of postoperative ICU validation studies, the applicability of ICE/AIS as primary sleep outcomes in immediate postoperative ICU care is uncertain, and current evidence supports their use mainly for characterizing pre-existing or persistent insomnia rather than short-term ICU-related sleep disturbance.

### 3.3 Psychometric assessment

Table 2 shows the results of the risk of bias evaluation concerning content validity for different tools, based on feedback from patients and experts. The review standards focus on how relevant, complete, and understandable patient assessments are. Experts looked at the relevance and completeness of these assessments. Patients found the PSQI tool very easy to understand and ranked it as acceptable on other factors. Patients gave the RCSQ a low grade for applicability, while experts ranked it very high. The SQQ showed steady performance with good scores in all areas. Patients found the ESS hard to understand, but experts ranked it very good for being relevant and thorough. The VSH instrument received mixed reviews. Patients rated it highly for being thorough but were unsure about how easy it was to understand. Patients ranked the ICE tools as very good for understanding, and they also gave good scores for other features.

**Table 2:** The Results of risk of bias (content validity).

Instruments	Content validity				
	Asking patients			Asking experts	
	Relevance	Comprehensiveness	Comprehensibility	Relevance	Comprehensiveness
PSQI	A	V	A	A	A
RCSQ	D	A	A	V	A
SQQ	A	A	A	A	A
ESS	D	A	I	V	V
VSH	A	V	D	A	A
ICE	V	A	V	A	A

Risk of bias rating: V, very good; A, Adequate; D, doubtful; I, inadequate.

Table 3 shows the results of an evaluation of the potential for bias in relation to a number of measurement characteristics for a variety of instruments. These attributes include criterion validity, reliability, structural validity, internal consistency, cross-cultural validity, and measurement error. Despite having questionable ratings for measurement error and criteria validity, the PSQI has satisfactory ratings for the majority of categories. Except for dependability, where it is deemed sufficient, the RCSQ receives extremely excellent ratings in the most other areas. The SQQ's reliability, measurement error, and criterion validity are good, however its cross-cultural validity is poor and its structural and internal consistency validity are uncertain. While the ESS received good scores in all other categories, its structural validity, internal consistency, and criteria validity were deemed dubious. Although the VSH received satisfactory scores in all other dimensions, its structural and cross-cultural validity were deemed dubious. With the exception of reliability, measurement error, and criteria validity, the ICE receives extremely excellent scores for the most of its features.

**Table 3:** The results of risk of bias (structural validity, internal consistency, cross- cultural validity, reliability, measurement error, criterion validity).

Instruments	Structural validity	Internal consistency	Cross-cultural validity	Reliability	Measurement error	Criterion validity
PSQI	A	I	A	A	V	V
RCSQ	V	V	V	A	V	V
SQQ	D	D	I	A	A	A
ESS	D	D	A	A	A	D
VSH	D	A	D	A	A	D
ICE	V	V	A	A	A	A

Risk of bias rating: V, very good; A, Adequate; D, doubtful; I, inadequate.

Table 4 shows the outcomes of the risk of bias evaluation for construct validity and responsiveness across many sleep-related measures. While responsiveness is examined by comparisons with other instruments, subgroup comparisons, and pre-post intervention changes, construct validity is evaluated depending on convergent validity, known groups validity, and comparison with a gold standard. The PSQI exhibits sufficient convergent validity for concept validity; it displays poor ratings for known groups validity and comparison with a gold standard elsewhere. Though regarded as sufficient when compared with a gold standard, the RCSQ has a dubious grade for both convergent validity and known groups validity. When contrasted with a gold standard, the SQQ is sufficient for convergent validity, questionable for known groups validity, and insufficient. The ESS has poor scores for comparability with a gold standard, known groups validity, and convergent validity. Though it is not as good as a gold standard, the VSH has sufficient ratings for known groups validity and convergent validity. Except for known groups validity in ICE, which is assessed as sufficient, the ICE exhibit quite strong scores across all three areas of construct validity.

**Table 4:** The results of risk of bias of construct validity and responsiveness.

Instruments	Construct validity		Responsiveness			
	Convergent validity	Known groups validity	Comparison with gold standard	Comparison with other instruments	Comparison between subgroups	Comparison before and after intervention
PSQI	A	V	I	I	I	A
RCSQ	D	D	A	A	A	A
SQQ	I	D	A	D	A	A
ESS	I	V	I	I	I	A
VSH	A	A	I	A	I	A
ICE	V	V	V	V	A	A

Risk of bias rating: V, very good; A, Adequate; D, doubtful; I, inadequate.

The PSQI is assessed as insufficient in comparisons with other instruments and subgroups but as acceptable in pre-post intervention comparisons for responsiveness. Except from a questionable assessment in comparisons with other instruments for SQQ, the RCSQ and SQQ get suitable ratings across all responsiveness criteria. Apart from pre-post intervention comparisons, where it is sufficient, the ESS gets low scores in all other areas. In subgroup comparisons the VSH is regarded as insufficient; in other respects, it is sufficient. While ICE gets very strong ratings for comparisons with other instruments and subgroups but is regarded as sufficient for pre-post intervention comparisons.

Table 5 presents the quality of evidence for the measurement properties of the PSQI, RCSQ, and SQQ instruments. Content validity is rated as moderate for both PSQI and RCSQ, while SQQ has a high rating. In terms of relevance, both PSQI and SQQ are rated as moderate, whereas RCSQ is rated low. Comprehensiveness is rated moderate for PSQI, high for both RCSQ and SQQ. Comprehensibility is rated moderate for PSQI and SQQ, but low for RCSQ. Structural validity is rated moderate for PSQI and RCSQ, while SQQ has a high rating. Internal consistency is low for PSQI, moderate for RCSQ, and high for SQQ. Cross-cultural validity is moderate for PSQI and SQQ, but high for RCSQ. Reliability is high for PSQI, moderate for both RCSQ and SQQ. Measurement error is rated low for PSQI and RCSQ, while moderate for SQQ. Criterion validity is very low for PSQI, moderate for RCSQ, and low for SQQ. Construct validity is moderate for PSQI and SQQ, but high for RCSQ. Responsiveness is rated low for PSQI and SQQ, but high for RCSQ.

**Table 5:** Quality of the evidence for measurement properties of the instruments.

Scale	PSQI		RCSQ		SQQ	
Measurement property	Rating	Qual. evid.	Rating	Qual. evid.	Rating	Qual. evid.
Content validity	+/–	Moderate	+/–	Moderate	+	High
Relevance	+/–	Moderate	+/–	Low	+/–	Moderate
Comprehensiveness	+/–	Moderate	+	High	+	High
Comprehensibility	+/–	Moderate	+/–	Low	+/–	Moderate
Structural validity	+/–	Moderate	+/–	Moderate	+	High
Internal consistency	+/–	Low	+/–	Moderate	+	High
Cross-cultural validity	+/–	Moderate	+	High	+/–	Moderate
Reliability	+	High	+/–	Moderate	+/–	Moderate
Measurement error	+/–	Low	+/–	Low	+/–	Moderate
Criterion validity	–	Very low	+/–	Moderate	+/–	Low
Construct validity	+/–	Moderate	+	High	+/–	Moderate
Responsiveness	+/–	Low	+	High	+/–	Low

Qual. evid., quality of evidence.

COSMIN rating definitions: “+”, sufficient; “+/–”, indeterminate; “–”, insufficient.

Table 6 presents the quality of evidence for the measurement properties of the VSH, ICE, and ESS instruments. Content validity is rated as moderate for VSH and ICE, while ESS has high-quality evidence. Relevance is rated as low for both VSH and ICE, whereas ESS has moderate quality. Comprehensiveness is low for VSH, moderate for ICE, and high for ESS. Comprehensibility follows a similar trend, with low ratings for VSH and ICE but high for ESS. Structural validity is consistently rated as moderate across all three instruments. Internal consistency is rated high for ICE and moderate for ESS, while VSH lacks a rating. Cross-cultural validity is rated as moderate for all instruments. Reliability is rated high for VSH and ESS but low for ICE. Measurement error is rated as moderate for VSH but low for ICE and ESS.

**Table 6:** Quality of the evidence for measurement properties of the instruments.

Scale	VSH		ICE		ESS	
	Rating	Qual. evid.	Rating	Qual. evid.	Rating	Qual. evid.
Content validity	?	Moderate	?	Moderate	+	High
Relevance	?	Low	?	Low	?	Moderate
Comprehensiveness	?	Low	?	Moderate	+	High
Comprehensibility	?	Low	?	Low	+	High
Structural validity	?	Moderate	?	Moderate	?	Moderate
Internal consistency			+	High	?	Moderate
Cross-cultural validity	?	Moderate	?	Moderate	?	Moderate
Reliability	+	High	?	Low	+	High
Measurement error	?	Moderate	?	Low	?	Low
Criterion validity	?	Moderate	?	Moderate	?	Moderate
Construct validity	?	Low	?	Moderate	?	Moderate
Responsiveness	?	Moderate	?	Moderate	?	Moderate

COSMIN Rating definitions: “+”, sufficient; “? ”, indeterminate; “– ”, insufficient.

## 4 Discussion

The present review included studies published between January 2010 and December 2024, thus the findings reflect the most recent decade of evidence available at the time of the search. Notably, an updated rapid scan of early 2025 publications did not identify any new ICU-focused validation studies for postoperative sleep instruments, indicating that psychometric research on these tools remains limited and that our synthesis captures the current state of knowledge. Within the 2010–2024 evidence base, the PSQI continues to be one of the most frequently used questionnaires across diverse clinical contexts, with multiple studies reporting acceptable internal consistency and stable test–retest reliability (Buysse et al., 1989; Al-Habsi et al., 2022; Chu et al., 2021). Nonetheless, mixed findings regarding criterion validity and its inconsistent correlation with objective sleep indicators, particularly in older adults raise concerns about the PSQI's suitability for postoperative ICU populations, who often experience rapid physiological fluctuations and medication-related sleep alterations (Wang et al., 2023). These limitations align with COSMIN observations that many widely used PROMs have not undergone population-specific validation.

In contrast, the Richards–Campbell Sleep Questionnaire (RCSQ) has shown stronger alignment with the needs of ICU research and practice. The tool has been validated across various cultural adaptations, including Chinese, German, Thai, Korea, Finland, Dutch, and other language versions, consistently demonstrating good internal consistency, structural coherence, and moderate-to-strong correlations with other subjective sleep measures (Jolley et al., 2018; Li S. B. et al., 2022; Ritmala-Castrén et al., 2022; Kim et al., 2020; Rood et al., 2015; Voiriot et al., 2022). However, findings are less consistent regarding criterion validity: several studies report low agreement between subjective RCSQ scores and objective measurements such as actigraphy or polysomnography (Locihová et al., 2018, 2020), and patient–nurse interrater reliability remains only mild to moderate, with nurses commonly overestimating sleep quality (Dorsch et al., 2019). These results highlight a persistent gap between subjective and objective assessments of postoperative ICU sleep as an issue that no instrument has fully resolved.

Sleep difficulties are therefore highly prevalent among hospitalized postoperative surgical patients, as multiple interacting variables, such as ambient noise, postoperative pain, medications, comorbidities, and intrinsic physiological factors disrupt both sleep continuity and depth (Kamdar et al., 2012). Poor sleep quality, particularly when accompanied by fragmentation, further contributes to delayed recovery, increased morbidity, and extended hospital stays (Boyko et al., 2017). Despite its recognized importance, sleep quality in postoperative surgical settings remains insufficiently examined, and the instruments used to assess sleep often demonstrate inconsistent performance or insufficient validation for this patient group. Understanding sleep quality in hospitalized postoperative patients is essential, as the interaction between the hippocampus and retrosplenial cortex during rapid eye movement (REM) sleep enhances memory recall through theta-driven connectivity (Dos Santos Bento et al., 2023). Moreover, slow-wave sleep plays a crucial role in memory consolidation, with slow oscillations and sleep spindles supporting the retention of newly acquired information (Scott et al., 2021). These neurophysiological mechanisms underscore the importance of accurately assessing postoperative sleep, as disturbances in sleep architecture can impair cognitive recovery and hinder the integration of new information during rehabilitation. Beyond cognitive consequences, sleep disturbance has been linked to a wide spectrum of adverse physical and psychological outcomes. Patients experiencing poor or fragmented sleep often report headaches, reduced satisfaction with life, and lower perceived general health (Lovato and Lack, 2019; Leilami et al., 2025). These effects can further compound postoperative vulnerability by diminishing emotional resilience, slowing mobilization, and reducing engagement in recovery activities.

The instrument aims to measure various aspects of sleep, including regularity and overall quality, and has shown promising psychometric properties in early validation studies. It shows a good fit with polysomnography and can effectively distinguish between good and poor sleep quality (Locihová et al., 2018). Other studies have also shown a moderate to strong correlation between the RCSQ and other sleep assessment tools, including actualography and polysomnography (Chen et al., 2022). However, some studies show low agreement between subjective RCSQ scores and objective measures of activity (Locihová et al., 2018). However, the reliability of patient-nurse interraters on RCSQ ranged from mild to moderate, with nurses tending to overestimate the quality of patients' sleep (Dorsch et al., 2019). While RCSQ moderately correlates with objective sleep measures such as actography and polysomnography, subjective sleep reports tend to overestimate the actual sleep duration Locihová et al. (2020).

ESS scores are significantly correlated with objective measures of sleep tendencies, such as the Multiple Sleep Latency Test (MSLT) (Amin et al., 2016). ESS showed a significant association with objective sleepiness as measured by the Multiple Sleep Latency Test (MSLT) when analyzed using survival analysis (Amin et al., 2016). However, ESS and MSLT are not interchangeable, because ESS is influenced by psychological factors that do not affect the outcome of MSLT (Cabassa and APNC, 2021). The ESS appears to measure the behavioral components of drowsiness, while other subjective measures assess internal state (Lewandowska et al., 2020). An ESS score of ≥13 optimally predicts objective sleepiness, which is higher than that typically used in clinical practice. ESS shows some correlation with polysomnographic parameters and sleep latency tests, but it is also influenced by psychological factors (Nilsen et al., 2020). In a clinical setting, ESS may be more useful for advanced assessment than initial screening, and should be supplemented with objective measures such as polysomnography and some sleep latency tests for an accurate diagnosis (Lim and Park, 2023).

Research on sleep and comprehensibility reveals complex relationships between sleep quality, language processing, and cognitive performance. The Verran and Snyder-Halpern (VSH) Sleep Scale is a reliable tool for measuring subjective sleep characteristics (Khoddam et al., 2022). Sleep-dependent consolidation of vocabulary in children with comprehension difficulties shows intact processes but weaker overall word memory (Shih et al., 2022). Hyperarticulated speech improves comprehensibility for both native and non-native listeners in various noise conditions (Jayanthi and Hudiyawati, 2019). Sleep quality in hospitalized patients is affected by environmental factors like light and noise (Chaudhary et al., 2020). Night shifts negatively impact vigilance, auditory attention, and comprehension, especially for less engaging or more complex material (Hemmati-Maslakpak et al., 2021; Lewandowska et al., 2020). Comprehensibility in L2 speech is influenced by various linguistic aspects, including lexical richness, fluency, grammar, and word stress (Amin et al., 2016).

ICE shows strong correlation with other sleep measures and health-related outcomes (Vlok, 2020). Factor analysis generally supports a two-factor structure for AIS-8 and a one-factor structure for AIS-5 (Al-Mughales et al., 2022). Factor analysis has favored the structure of one factor and two factors, with the latter distinguishing between nocturnal symptoms and daytime consequences (de la Varga-Martínez et al., 2023). The limit value for detecting insomnia has been set in different populations (Chung et al., 2023). The AIS, along with other sleep quality measures such as the Insomnia Severity Index, has been recognized as a reliable and valid tool for assessing sleep quality across a variety of clinical settings and research (Chen et al., 2016). AIS has shown usefulness in detecting insomnia and depression in diverse populations (Suen et al., 2019), making it a valuable screening tool for sleep disorders in a variety of clinical settings.

## 5 Conclusion and recommendation

This systematic review provides an integrated evaluation of the psychometric properties of sleep assessment instruments used among hospitalized postoperative surgical patients, including those in intensive care, high-dependency, and general surgical units. Using the COSMIN framework, the review highlights that while commonly used tools such as the PSQI, RCSQ, SQQ, ESS, and AIS demonstrate varying degrees of reliability, validity, and feasibility, no single instrument exhibits consistently strong measurement performance across all COSMIN domains. Among the identified tools, the RCSQ shows the most robust evidence for use in ICU and high-acuity environments, offering strong reliability and practical applicability for assessing postoperative sleep quality. The PSQI remains a widely adopted general sleep measure, yet its inconsistent factor structure, limitations in measurement error, and variable responsiveness indicate the need for cautious interpretation in postoperative populations. Other instruments, including the SQQ, ESS, and AIS provide useful complementary insights but are not sufficient as standalone measures for capturing the multidimensional sleep disturbances common in postoperative and critically ill patients. Overall, the findings underscore the need for developing and validating sleep assessment tools specifically tailored to the postoperative and ICU context, where sleep fragmentation, environmental disruptions, and acute physiological stressors are prominent. Future research should prioritize rigorous psychometric testing, cross-cultural validation, and the incorporation of clinically meaningful outcomes to strengthen the accuracy and utility of sleep assessment in these vulnerable patient populations.

## Data Availability

The datasets presented in this study can be found in online repositories. The names of the repository/repositories and accession number(s) can be found in the article/Supplementary material.
