# Supplementary material for: Sleep quality assessment in hospitalized postoperative surgical patients: a COSMIN-based systematic review
Source: Front Sleep. 2026 Apr 13;4:1714777. doi: 10.3389/frsle.2025.1714777 (PMC13111034; doi:10.3389/frsle.2025.1714777)
Supplement: Supplementary file 1 [file Table_1.docx]

# **Supplementary Table S1. Clinical Setting Classification of Included Studies (ICU, Mixed, or Surgical Ward)**

| **No.** | **Study (Author, Year)** | **Instrument(s)** | **Population Setting** | **ICU Relevance Note** |
| --- | --- | --- | --- | --- |
| 1 | Seid Tegegne & Fenta Alemnew (2022) | PSQI | Surgical ward | Not ICU; general postoperative patients |
| 2 | Selçuk Sert et al. (2024) | PSQI | Surgical ward | Not ICU |
| 3 | Prakrithi et al. (2019) | PSQI | Surgical ward | Not ICU |
| 4 | Yilmaz et al. (2012) | PSQI | Surgical ward | Not ICU |
| 5 | Büyükyilmaz et al. (2011) | PSQI | Surgical ward | Not ICU |
| 6 | Barichello et al. (2009) | PSQI | Surgical oncology ward | Not ICU |
| 7 | Telias & Wilcox (2019) | PSQI | Post-ICU follow-up | ICU survivors; relevant |
| 8 | Solverson et al. (2016) | PSQI | Post-ICU survivors | ICU-related sleep quality |
| 9 | Oren et al. (2020) | PSQI | Surgical ward (thoracic) | Not ICU |
| 10 | Leong et al. (2021) | PSQI | Surgical ward | Not ICU |
| 11 | Okkesim et al. (2019) | PSQI | Surgical ward (hand surgery) | Not ICU |
| 12 | Yıldız et al. (2023) | PSQI | Surgical ward | Not ICU |
| 13 | Chen et al. (2016) | PSQI, ESS | Orthopedic surgical ward | Not ICU |
| 14 | Luo et al. (2019) | PSQI, ESS | Orthopedic surgical ward | Not ICU |
| 15 | Bang & Park (2020) | PSQI, ESS | Cardiac surgery ward | Not ICU |
| 16 | Grubbs et al. (2023) | PSQI, RCSQ | ICU | ICU patients |
| 17 | Díaz-Alonso et al. (2018) | PSQI, RCSQ | ICU | ICU patients |
| 18 | Wang et al. (2019) | PSQI, RCSQ | ICU | ICU patients |
| 19 | Hu et al. (2022) | PSQI, ESS | Surgical ward (cardiac infection surgery) | Not ICU |
| 20 | Navarro-García et al. (2017) | RCSQ | ICU | ICU patients |
| 21 | Allen et al. (2022) | RCSQ | Surgical ward | Not ICU |
| 22 | Rood et al. (2019) | RCSQ | ICU | ICU patients |
| 23 | Nagatomo et al. (2020) | RCSQ | ICU | ICU patients; device validation |
| 24 | Tonna et al. (2021) | RCSQ | Surgical ICU | ICU patients |
| 25 | Alsulami et al. (2019) | RCSQ | ICU | ICU patients |
| 26 | Fazlollah et al. (2021) | RCSQ | Cardiac surgery ward | Not ICU |
| 27 | Myoji et al. (2015) | SQQ | Orthopedic surgical ward | Not ICU |
| 28 | Bakry et al. (2022) | ESS | Cardiac surgery ward | Not ICU |
| 29 | Khoddam et al. (2022) | VSH | Coronary care unit (CCU) | Critical care but not ICU |
| 30 | Li et al. (2021) | ICE | ICU | ICU patients |
| 31 | Varella et al. (2021) | RCSQ | ICU | ICU patients |
| 32 | Locihová et al. (2020) | RCSQ, Actigraphy | ICU | ICU patients |
| 33 | Delaney et al. (2022) | Actigraphy, RCSQ | ICU | ICU patients |
| 34 | Jayanthi & Hudiyawati (2019) | Sleep therapy evaluation | ICU | ICU intervention study |
| 35 | Ritmala-Castren et al. (2022) | RCSQ | ICU + home sleepers | ICU subgroup relevant |
| 36 | Lewandowska et al. (2020) | Qualitative ICU sleep study | ICU | ICU-specific |
| 37 | Nagatomo et al. (2020) | Under-mattress sleep sensor | ICU | ICU validation study |
